# A Dangerous Detour: Invasive Salmonella in a Healthy Traveler

**DOI:** 10.7759/cureus.90316

**Published:** 2025-08-17

**Authors:** Calvin Yee Fen Lee, Ayra Kathuria, Aliza Yaqub, Sissmol Davis, Ngoc Thai Kieu

**Affiliations:** 1 Internal Medicine, University at Buffalo Jacobs School of Medicine and Biomedical Sciences, Buffalo, USA

**Keywords:** bacteremia, immunocompetent adult, invasive infection, non-typhoidal salmonella, traveler's diarrhea

## Abstract

Traveler’s diarrhea is a common self-limited illness among international travelers, typically caused by enteric pathogens such as *Escherichia coli*, *Campylobacter*, and non-typhoidal *Salmonella *(NTS). Invasive NTS infections, including bacteremia, are rare in immunocompetent adults. We report a case of *Salmonella *bacteremia in a previously healthy 30-year-old man who presented with persistent watery diarrhea and fever after returning from travel to the Philippines and Japan. Despite lacking traditional risk factors for invasive NTS disease, blood cultures grew non-typhoidal *Salmonella *spp. three days after initial discharge. The patient required hospitalization and treatment with intravenous ceftriaxone followed by oral trimethoprim-sulfamethoxazole, with full resolution of symptoms. This case highlights that invasive *Salmonella *infections can occur even in young, immunocompetent individuals without apparent high-risk exposures. Clinicians should maintain a high index of suspicion for invasive enteric pathogens in travelers with persistent symptoms, as prompt diagnosis and treatment are critical to preventing complications.

## Introduction

Traveler’s diarrhea is one of the most common ailments among international travelers. It is usually self-limited and typically caused by enteric pathogens such as *Escherichia coli*, *Campylobacter*, and non-typhoidal *Salmonella *(NTS). Invasive infections caused by NTS are rarely seen in healthy adults. Only an estimated 1-4% of enteric NTS infections progress to bacteremia, with an annual incidence of culture-confirmed invasive *Salmonella* of approximately 0.9 per 100,000 in the United States [1].

Progression to invasive disease is more frequently observed in individuals with identifiable risk factors such as immunosuppression, extremes of age, chronic comorbidities (including HIV, malignancy, or diabetes), or recent antimicrobial use. In the absence of these factors, infection is typically limited to the gastrointestinal tract. Current guidance recommends that when invasive NTS is suspected, such as in the setting of persistent fever, focal symptoms, or systemic toxicity, evaluation should include blood cultures in addition to stool cultures, and consideration of targeted investigations to assess for extraintestinal foci of infection (e.g., endovascular or osteoarticular involvement).

Here, we present a rare case of *Salmonella *bacteremia in a young, previously healthy traveler, highlighting an uncommon but potentially serious outcome of what initially appeared to be routine traveler’s diarrhea.

## Case presentation

A 30-year-old man with no significant medical history presented to the emergency department with a 10-day history of persistent watery diarrhea and a two-day history of fevers, with a reported maximum temperature of 101.8°F. He had returned from international travel to the Philippines and Tokyo two weeks earlier, with gastrointestinal symptoms such as loose watery diarrhea and generalized abdominal pain beginning during his stay in the Philippines. He denied high-risk food exposures, reported traveling in groups, and drank only filtered water. He denied chest pain, palpitations, dyspnea, nausea, vomiting, urinary symptoms, scrotal swelling, penile discharge, rashes, or any known sick contacts.

**Figure 1:**
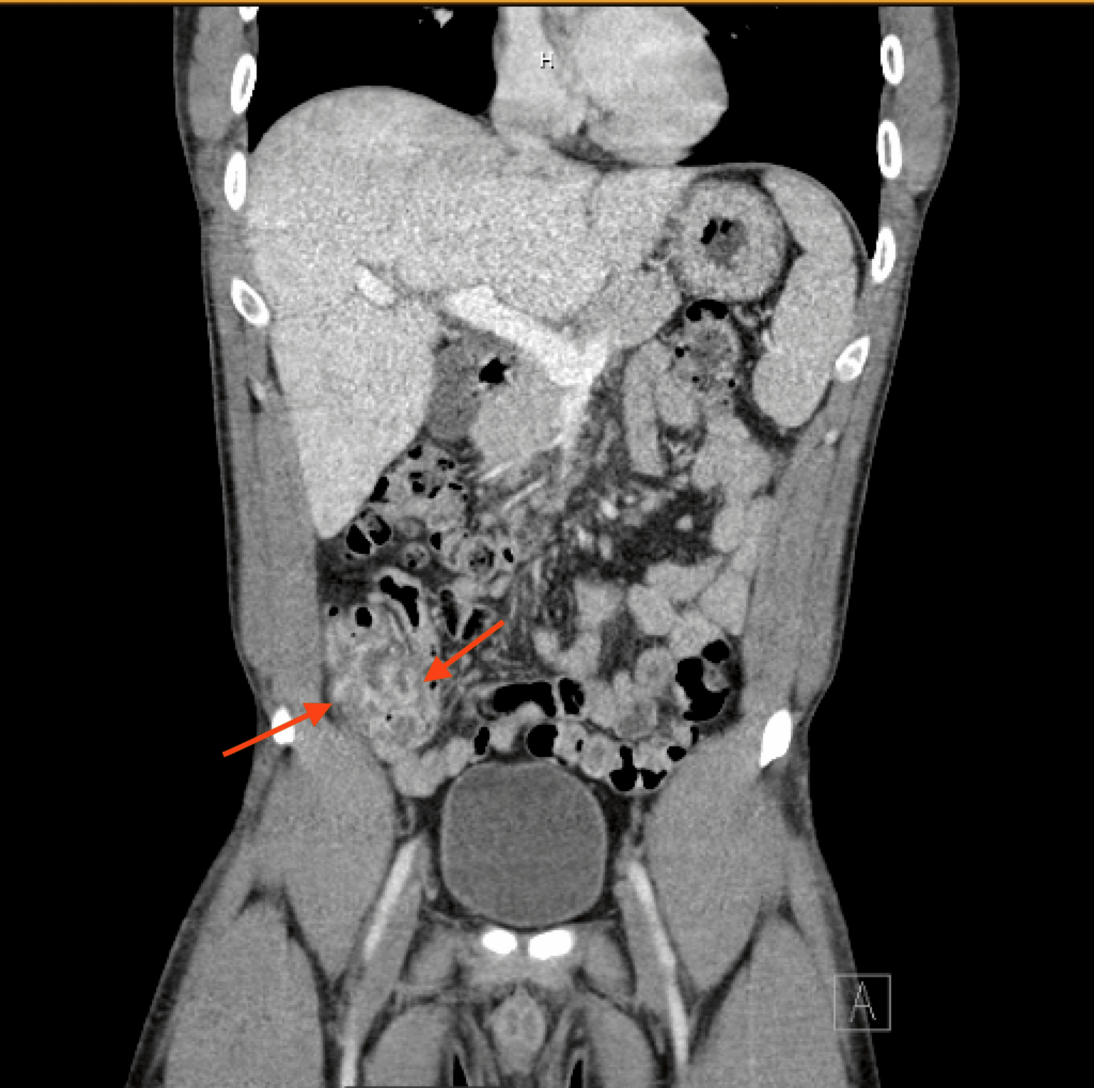
CT abdomen and pelvis showing mild colitis Image of CT abdomen and pelvis showing mildly thickened cecum/ascending colon walls (marked with red arrows), suggestive of colitis

In the emergency department, he was afebrile with a heart rate of 64 beats/minute, a respiratory rate of 20/minute, blood pressure of 114/81 mm Hg, and oxygen saturation of 98% on room air. Physical examination was notable for diffuse abdominal tenderness without peritoneal signs. On initial investigations, he tested positive for SARS-CoV-2. Complete blood count showed hemoglobin 13.9 g/dL, WBC 5.1 × 10^3^/µL, and platelets 181 × 10^3^/µL. The comprehensive metabolic panel was unremarkable. Lipase and lactic acid were within normal limits. Urinalysis was unremarkable, and urine toxicology was positive for cannabinoids. HIV, hepatitis B, and hepatitis C testing were non-reactive. A CT of the abdomen demonstrated mild thickening of the cecum and ascending colon (Figure 1), suggestive of colitis. Blood cultures were obtained, and the patient was discharged with a presumptive diagnosis of traveler’s diarrhea and prescribed oral amoxicillin-clavulanate.

Three days later, he was recalled after blood cultures grew non-typhoidal *Salmonella*. Upon readmission, he was afebrile, with a heart rate of 62 beats/min, a respiratory rate of 17/min, blood pressure of 115/75 mm Hg, and oxygen saturation of 98% on room air. He was admitted and initiated on intravenous ceftriaxone 2 g daily. Stool cultures obtained on day 2 showed no growth. Stool ova and parasites, as well as shiga toxin and shiga-like toxin, were negative. Intravenous ceftriaxone was transitioned to oral trimethoprim-sulfamethoxazole (800 mg/160 mg) on day 3, which was continued for 8 more days. His symptoms resolved fully by day 4, and he was discharged.

## Discussion

This case illustrates that even in young, otherwise healthy adults, NTS* *can cause severe invasive disease. While NTS is a common cause of self-limited gastroenteritis, responsible for approximately 1.35 million infections annually in the United States, progression to bacteremia is rare and typically associated with identifiable risk factors such as immunosuppression, extremes of age, or chronic comorbidities [2,3]. The absence of such risk factors in our patient suggests that a large inoculum or a particularly virulent strain may have been acquired abroad. His clinical course underscores the need to maintain a high index of suspicion for invasive enteric infections in returning travelers, even those without traditional risk factors.

Globally, invasive NTS* *(iNTS) represents a substantial and often underrecognized public health concern. Although invasive disease is rare in high-income countries, it remains a leading cause of bacteremia and death in low- and middle-income regions, particularly in sub-Saharan Africa, where case fatality rates can reach 20% to 25% [4]. An estimated 3.4 million cases of iNTS and more than 680,000 associated deaths occur worldwide each year, disproportionately affecting populations with limited access to healthcare and comorbid conditions such as HIV, malaria, and malnutrition [5]. These global patterns emphasize that the burden of iNTS extends far beyond what is typically seen in the United States, and that international travel can serve as a conduit for exposure to more aggressive or resistant *Salmonella *strains.

Travel-associated *Salmonella *infections are well documented. One study found that among 23,712 *Salmonella*-positive case-patients, 11% had traveled internationally in the seven days before illness [6]. Such infections are more likely to lead to hospitalization and invasive complications than domestically acquired cases. Our patient’s travel to the Philippines, a region with higher NTS prevalence and potential for multidrug-resistant strains, may have contributed to his more severe presentation. In such cases, early recognition and appropriate antimicrobial therapy are critical to prevent complications such as endovascular seeding, abscess formation, or prolonged bacteremia. The treatment options for invasive *Salmonella *bacteremia are parenteral third-generation cephalosporins (such as ceftriaxone) or fluoroquinolones (such as ciprofloxacin), with the choice guided by local resistance patterns and susceptibility testing. Trimethoprim-sulfamethoxazole or amoxicillin may be used if the isolate is susceptible, but these are not first-line empiric agents due to widespread resistance. Our patient was treated with first-line intravenous ceftriaxone, which was later transitioned to oral trimethoprim-sulfamethoxazole as the organism was sensitive to trimethoprim-sulfamethoxazole.

From a public health perspective, invasive NTS not only poses a threat to the individual but also carries the potential for broader transmission, particularly among food handlers, healthcare workers, and immunocompromised contacts. Long-term prevention strategies must include continued investment in water and food safety infrastructure globally, especially in travel destinations with known endemicity. Moreover, vaccine development targeting the most common invasive serotypes such as *S. typhimurium* and *S. enteritidis* is underway and may play a critical role in reducing global disease burden [4].

In the interim, clinicians should educate travelers on preventive measures, including avoiding high-risk foods, using safe water sources, and seeking prompt care for persistent or systemic symptoms. As global travel continues to increase, the risk of importing virulent or resistant enteric pathogens into low-incidence settings will rise. This case illustrates how common travel-related illnesses can mask serious systemic infections, reflecting the dynamic interface between clinical medicine and global health.

## Conclusions

With the growing volume of international travel, clinicians in low-incidence settings must remain vigilant for atypical infections such as invasive salmonellosis. Recognizing and treating *Salmonella *bacteremia early is vital, as delays can lead to complications including abscesses or vascular involvement. This case highlights the importance of maintaining a high index of suspicion for invasive enteric pathogens in returning travelers with persistent symptoms, regardless of immune status.
